# A study on the effect of different channel cues on learning in immersive 360° videos

**DOI:** 10.3389/fpsyg.2024.1335022

**Published:** 2024-04-17

**Authors:** Guan Huang, Chao Chen, Yahan Tang, Haohua Zhang, Rui Liu, Li Zhou

**Affiliations:** College of Education, China West Normal University, Nanchong, China

**Keywords:** 360°video, cues, eye movement test, brainwave test, learning result

## Abstract

Immersive 360° videos are of interest to educators because of their ability to provide immersive sensory experience and other features. This study examined the effects of four cue conditions on 360° video learning performance, attention, cognitive load, and mood using eye-tracking devices, brainwave meters, and subjective questionnaires. The randomly assigned participants (*n* = 62) did go to the experimental group (visual cues only, auditory cues only, and audiovisual cues) or the control group (no cues). The results showed that visual and audiovisual cues effectively guide learners’ attention to the related learning content, reduce cognitive load during learning, and improve retention performance but have no significant effect on knowledge transfer or long-term memory. Auditory cues increase the number of times learners look at the related learning content but do not affect gaze duration and distract their attention, hindering the acquisition of relevant learning content. The study also found that visual cues effectively increase the number of times learners looked at the content. However, they do not affect gaze duration. The study also revealed that visual cues effectively increase learners’ relaxation when viewing 360° videos. The study’s findings can provide a reference for the instructional processing of information related to 360° video design and its practical application in teaching.

## 1 Introduction

With its immersive presence and interactive features (Albus et al., 2021), the deep integration of virtual reality technology with education brings more possibilities for learning. For example, virtual reality-based learning can increase the sense of presence in the learning process (Makransky et al., 2019). Studies have shown that virtual reality can effectively improve learning outcomes (Merchant et al., 2014). Virtual reality is split into virtual desktops, cave virtual environments, and immersive virtual reality with head-mounted displays (HMDs), which can be accessed differently (Buttussi and Chittaro, 2018).

360º videos have good applicability; they not only can be viewed on computers, mobile phone tablets, VR boxes, and HMDs but also have the advantages of ease of use and low cost (Shadiev and Sintawati, 2020). 360° videos have received attention due to their high potential for application in education. The immersive 360° video discussed in this study is a panoramic video viewed through a dedicated virtual reality display (HMD) device (e.g., HTC Vive, Oculus Rift), which enhances immersion in the learning process (Harrington et al., 2018), allowing viewers to turn their heads to view the video as if they were exploring the real world. As 360° videos involve 360 visual angle visible scene pictures and linearly changing audio commentary, learners may experience distraction or difficulty concentrating on the learning process, thus causing learners to ignore important learning content, affecting the necessary cognitive processing, and ultimately reducing their academic performance. Therefore, the question of how to guide learners’ attention to the necessary content in 360° video immersive learning environments is worth further study.

The use of cues in traditional media can direct learners’ attention to relevant material and thus increase necessary processing and reduce the external processing of irrelevant material (Mayer and Fiore, 2014), reducing cognitive load (Moreno and Abercrombie, 2010). Studies have provided reference cases for solving the above problems by guiding learners in 360° videos. Therefore, this study investigated whether the inclusion of cueing in immersive 360° videos affects learners’ attention, cognitive load, and learning performance. This research can help guide the design and development of 360° educational videos (e.g., educators who want learners to focus on a necessary piece of information can add cues to direct their attention and promote understanding of the relevant information).

### 1.1 360° video in education

In contrast to the flat screen of traditional video, 360° video is immersive and spherical (Moreno and Abercrombie, 2010), creating an almost realistic learning environment (Parmaxi, 2020). Importantly, unlike VR, 360° videos do not allow users to interact with elements in the virtual environment. However, learners can stand at a point of view and view the video content left, right, up, and down (Ward, 2017), creating the illusion of place and an immersive experience that increases learner engagement (Harrington et al., 2018) and improves learning outcomes (Rupp et al., 2019). 360° videos have been successfully used in various educational fields, including medicine (Yoganathan et al., 2018), sports (Liu et al., 2020), science (Wu et al., 2019), and lecture training (Stupar-Rutenfrans et al., 2017). For example, Yoganathan et al. (2018) demonstrated that 360° videos help medical students acquire knot-tying skills; scholars such as Stupar-Rutenfrans et al. (2017) confirmed that learners can effectively relieve nervousness about speaking when 360° videos represent a natural environment; and Araiza-Alba et al. (2020) used 360° videos, traditional videos, and posters to teach water safety skills and found that learners considered 360° videos to be more inspiring and attractive than traditional learning methods. However, the impact of 360° videos on learners is not always positive. Rupp et al. (2016) found that 360° videos made learners focus more on the immersive experience than on the learning content, resulting in distractions and even increased cognitive load, hindering the processing of relevant knowledge (Licorish et al., 2018). They drew on previous research findings in 2D media: the more significantly the total cognitive load was reduced by the cue, the better the retention and transfer of multimedia learning was (Xie et al., 2017). The research conclusions enlighten us about the need to understand the effects of cues in 360° videos and whether they can address the issues of guiding learners to select specific information for processing, reducing cognitive load, and providing learning effects in 360° videos.

### 1.2 The cognitive process in 360° videos

The learning content in 360° videos is presented to learners through narration and visual images. Based on Baddeley’s working memory model, cognitive resources can be best used when visual information is simultaneously presented in an auditory manner (Baddeley, 1992). The cognitive theory of multimedia learning (CTML) (Mayer, 2005a,b) considers selection, organization, and integration to be meaningful learning processes. Selection is achieved through an attentional process. The all-encompassing visibility of 360° video makes the selection process more complex because learners must filter out more elements that are irrelevant to learning (Albus et al., 2021). Moreover, the capacity of each channel of the cognitive system is limited, and the learner’s ability to organize and process information is limited (Sweller et al., 1998). Therefore, the information presented in 360° videos may lead to information overload, increasing cognitive load during learning and affecting the learner’s processing and integration of learning content.

### 1.3 Principle of multimedia learning cues

Cueing is an instructional design approach in which noncontent information is used in multimedia learning to draw learners’ attention to critical information and promote learning achievement (De Koning et al., 2007, 2009). Mayer, (2005a,b) divided cues into visual and verbal cues, where visual cues include colors, spotlights, arrows, etc., and verbal cues include titles, outlines, etc. According to the multimedia learning principles proposed by Mayer (2014), cues can provide good support for learners’ complex selection, processing, and integration processes in learning through 360° video content. Previous studies in 2D media have shown that cues can direct learners’ attention to specific learning content (Mayer, 2005a,b), reducing the time spent visually searching for less relevant areas (Jamet, 2014) and improving learning performance (Grant and Spivey, 2003). However, scholars disagree over whether cues are still relevant in immersive virtual reality environments. Cooper et al. (2018) suggested that multisensory feedback cues effectively provide additional information to improve overall task performance and user-perceived presence. Pizzoli et al. (2021) reported that olfactory cues can trigger distant memories, reduce the cognitive load required to re-evoke memories and experiences, and help learners relax. However, other studies have shown no significant effect of cues on cognitive load (Albus et al., 2021) or posttest performance (Hwang and Shin, 2018) for learning in virtual reality environments. In addition, scholars in other fields have explored the weighting of different sensory cues on individual influences. Soares et al. (2021) analyzed the role of visual and auditory cues in pedestrian crossing decisions and found that pedestrian crossing decisions were based mainly on pedestrians’ visual perceptions of the motion characteristics of approaching vehicles. Keshavarz et al. (2017) explored the effect of different cues on the estimated time-to-contact (TTC) in a virtual reality environment and found that visual cues were generally dominant. However, there are no relevant findings in the educational domain.

Based on the above analysis, given that learners must perform the complex processes of selecting, organizing, and integrating information in VR with limited working memory, thought should be given to how cues can be better designed to be embedded in 360° videos to support learners. This study refers to Paivio’s (1991) work, where cues act on visual, auditory, and audiovisual channels. Therefore, this study’s dual encoding theory uses eye-tracking devices, brainwave meters, and questionnaires to assess how cues acting on different channels affect 360° video learning.

## 2 Hypothesis

This study aimed to investigate the effects of different types of cues (visual, auditory, and audiovisual channels) embedded in 360° videos on learning performance, attention, emotion, and cognitive load in 360° video learning.

*Hypothesis 1*: Cues effectively guide learners to learn the visual attention distribution of 360° videos. The frequency of visual attention allocation to relevant learning content is higher for audio-visual cues than for visual cues and lowest for auditory cues.

*Hypothesis 2*: Cues effectively regulate learners’ implicit attention allocation when learning content with 360° videos. The frequency of visual attention allocation to relevant learning content is higher for audio-visual cues than for visual cues and lowest for auditory cues.

*Hypothesis 3*: Cues reduce the cognitive load of learners learning using 360° videos, and the cognitive load of audiovisual cues is lower than that of visual and auditory cues.

*Hypothesis 4*: Cues enhance learners’ learning performance in 360° videos, and the visual learning performance of the audiovisual cue group is better than that of the visual and auditory cue groups.

## 3 Methodology

### 3.1 Participants

This study randomly recruited more than 90 undergraduate and graduate students from majors such as Chinese Language and Literature, Education, and Foreign Language and Literature from a certain normal university as candidates. The selection criteria were high school students in liberal arts or not taking biology in the New College Entrance Examination and who passed the cell structure knowledge test (excluding participants with scores exceeding 6 points). Finally, this study selected sixty-two participants as valid; these included seven males and 55 females aged between 19 and 24 years (*M =* 20.42, *SD* = 1.139). With the consent of the participants, researchers randomly divided them into four groups and paid a 15 yuan labor fee after the experiment.

### 3.2 Experimental devices

This study used the HTC VIVE Pro Eye eye-tracking device to record the participants’ eye movement data with a monocular resolution of 1,440 × 1,600 and a binocular resolution of 3 K (2,880 × 1,600). The refresh rate was 90 Hz, the field of view was 110 degrees maximum, the participants maintained a standing position during the experiment, and 5 points were used for eye movement calibration in the experiment.

The BrainLink Pro brainwave instrument, a Macro Intelligence Technology Co., Ltd. product, was used as a portable brainwave instrument. The present study used brain data derived from brainwave instrument concentration, relaxation, and brain use data. Attention measured by the brainwave instrument during learning process reflects the concentration level of the brain in real time, with 0 being the lowest and 100 being the highest. Importantly, attention measured by brainwaves is different from attention measured by eye tracking in terms of the number of gaze sessions and average gaze duration. Wright and Ward (2008) classify attention into two types: externally explicit and implicit. Extrinsic attention is an individual’s response to external stimuli, such as visual attention. Implicit attention, which is the process of internally enhancing neural processing of signals received by the sensory apparatus, is usually not directly externally observable. Therefore, the attention measured by brainwaves here is implicit. Brain use reflects the brain’s real-time workload; the higher the brain workload is, the higher the value, with-100 being the lowest and 100 being the highest. Relaxation indicates the degree of relaxation of the brain. The more relaxed the brain is, the lower the value is; 0 is the lowest, and 100 is the highest.

Brainwaves are electrical changes in nerve cells and neuronal pulses in the brain, and the current principles of brain electricity generation involve mainly action and postsynaptic potentials. Modern scientific research has shown that the brain produces electrical signals when it works, dividing them into gamma waves, delta waves, theta waves, alpha waves, and beta waves according to the frequency range (Abhang et al., 2016). The characteristics of the five basic EEG waves are shown in Table 1.

**Table 1:** Characteristics of the five basic brainwaves (Abhang et al., 2016).

Frequency band	Frequency	Brain states
Gamma (γ)	35 Hz	Concentration
Beta (β)	12–35 Hz	Anxiety dominant, active, external attention, relaxed
Alpha (α)	8–12 Hz	Very relaxed, passive attention
Theta (θ)	4–8 Hz	Deeply relaxed, inward focused
Delta (δ)	0.5–4 Hz	Sleep

In this study, brainwave data were collected while the participants were viewing 360° videos with the help of a brainwave instrument. The brainwave device used in this study collected data while ensuring that the participants wore and used the HTC VIVE HMD. The α-wave β-wave δ-wave, γ-wave and θ-wave EEG signals recorded the brainwave data from the learning process and calculated the learner’s concentration, relaxation, and brain use through built-in algorithms. Its basic EEG parameters include a dry electrode contact sensor, with a collection frequency of 3–100 Hz, a sampling rate of 512 Hz, a bandwidth of 100 Hz, an ADC of 24 bits, a maximum input impedance of 20 Mohm, and a signal transmission method for the serial port (UART). This brainwave instrument has been successfully used in previous studies (Lin and Li, 2018; Phanichraksaphong and Tsai, 2019).

### 3.3 Experimental materials

All the participants were randomly divided into four groups, with 16 participants watching the A (no cues) video, 16 watching the B (visual cues only) video, 15 watching the C (auditory cues only) video, and 15 watching the D (audiovisual cues) video. To control for the influence of other external variables on the experiment, four groups of participants participated in the experiment strictly according to the experimental procedure.

#### 3.3.1 Video

The 360° video resource was taken from the YouTube platform The Body VR: Journey Inside a Cell, which was successfully used in a study by Meyer et al. (2019). The 360° video is broadly based on a virtual environment in which the participant travels in a cellular space within the bloodstream, crosses blood vessels to reach an individual cell in the tissue fluid and enters it, and then observes the internal structure and how the cell works. First, according to the experimental needs, we translated and dubbed the 360° video resources and invited two first-line high school biology teachers to correct the content and make appropriate corrections to the 360° video resources. The final video length was 7 min and 21 s.

#### 3.3.2 Audio

All audio in the 360° video was recorded in a professional recording studio using a panoramic camera and dubbed by a student majoring in broadcasting and hosting. The audio produced was rendered as stationary flat stereo rendered audio. Noise reduction, volume level and timbre were processed through Adobe Audition software, and the audio output format was stereo. The edited video and audio were synthesized through the VR function module in Adobe Premiere Pro software. The subjects watched and listened to the experimental material through the HMD device HTC VIVE (which supports stereo playback).

Finally, the 360° video was processed in to versions without any cues, with visual cues, with auditory cues, and with audiovisual cues. The visual cues were presented with arrows and text only at appropriate locations, and the auditory cues were presented only by voice narration. Audiovisual cues involve the simultaneous presentation of both the visual and auditory information mentioned above. Examples of the auditory and visual cues are shown in Table 2.

**Table 2:** Examples of auditory and visual cues.

Scene screenshot	Audio content	Visual cues	Auditory cues
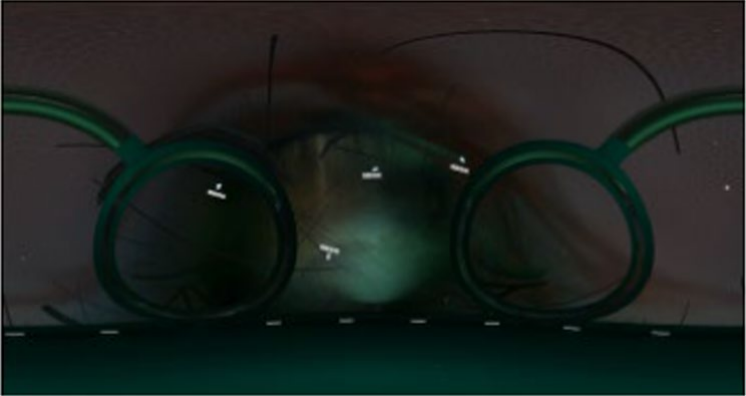	The network structure we see is a skeleton composed of microfilaments, intermediate filaments, and microtubules. Microfibers are solid fibers with a diameter of only 7 nanometers, mainly used for support, non muscular movement, and information transmission.	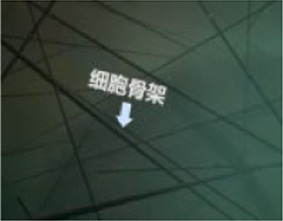	“Around the spacecraft”
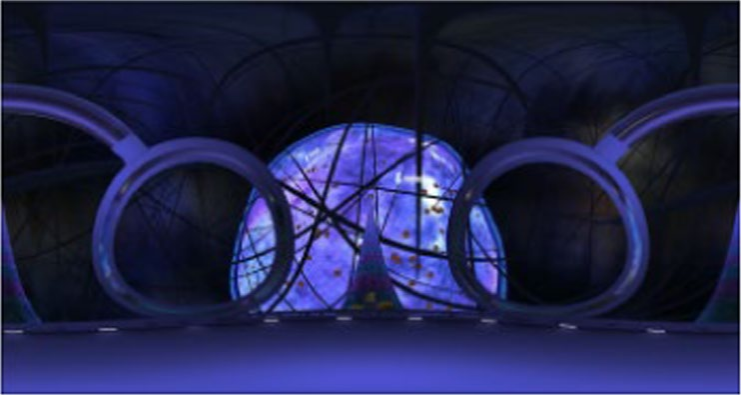	We can see that the surface of the nucleus has its own membrane, similar to the cell membrane. The nuclear membrane completely surrounds the nucleus, separating the genetic material of the cell from the surrounding cytoplasm, serving as a barrier to prevent the free diffusion of large molecules between the nucleus and cytoplasm.	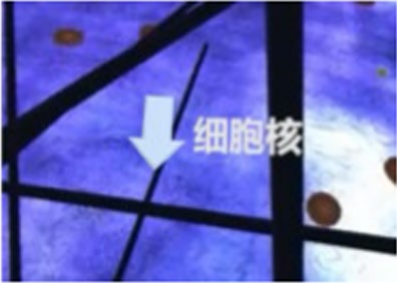	“In front of the spacecraft”
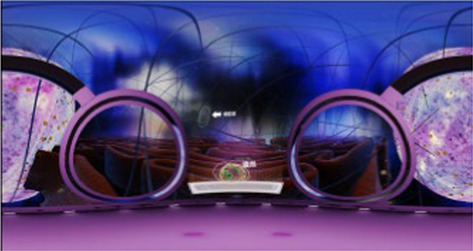	You can see a floating mitochondria, the main site for aerobic respiration in cells, providing energy for various life activities, so mitochondria are often referred to as the power factory of cells.	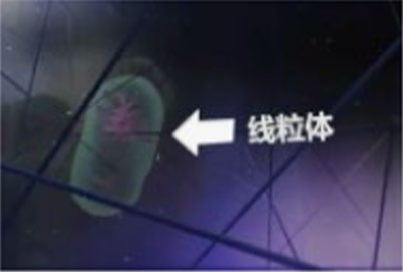	“In the direction of the spacecraft’s advance”

Due to the fact that the entire 360° video simulates the process of a learner traveling the entire cell in a spaceship, and the relative position between the learner and the spaceship remains unchanged, this study provides voice prompts to the learner by describing the position relationship with the spaceship. Images adapted with permission from The Body VR, available at The Body Vr, “The Body VR: Journey Inside a Cell - 360 Gameplay”, YouTube video, 10:35, 23 August 2016, https://www.youtube.com/watch?v=9zTsDXMyBEY.

#### 3.3.3 Prior knowledge test

The questions were based on a pretest-test questionnaire used in Mayer’s Media and Methods experiment and modified by two first-line high school biology teachers to measure the participants’ knowledge of cells.

#### 3.3.4 Emotion measurement

Subjective emotion was measured using Watson et al.’s (1988) Positive Affect Questionnaire to measure changes in participants’ emotions before and after the experiment and was scored on a 5-point Likert scale. The reliability coefficients of the pre-and post-scale measures were 0.881 and 0.858, respectively.

#### 3.3.5 Cognitive load measurement

Sweller et al. (2019) classified cognitive load into intrinsic, external, and associative loads. Frederiksen et al. (2020) discussed the relationship between being in a virtual reality environment and the three types of cognitive load. Therefore, this study adopted Paas’s (1994) Subjective Evaluation Scale and Leppink et al., (2013) Cognitive Load Scale to develop a cognitive load questionnaire. The questionnaire includes an evaluation of the 360° video materials’ learning task difficulty, subjective psychological effort, internal cognitive load, external cognitive load, and associated cognitive load. The items are scored on a 9-point Likert scale. The reliability coefficients of the subjective mental effort scale and cognitive load scale were 0.692 and 0.700, respectively.

#### 3.3.6 Attention measurement

Attention is a human mental activity that is part of the cognitive process. In this study, episodic measures of visual attention were made by eye-tracking technology, and studies have shown that visual attention is correlated with gaze (Pi et al., 2019). In this study, the number of gazes and the average gaze duration when cues appeared in seven scenes of the 360° video were selected to examine the visual attention allocation of subjects. The time spent on the first gaze in each scene’s area of interest was also selected as the effect of the cue on the visibility of the learning content.

#### 3.3.7 Learning achievement test

In this study, the learning achievement test included a retention test, migration test, and knowledge forgetting test. In the retention test, 16 single-choice questions related to the content of the 360° video were presented, for a score of 16 points. In the migration test, the ability of the participants to apply the cellular knowledge learned from the 360° video to new situations was investigated, and three subjective questions were set, each with 3 points and a total possible score of 9 points. In the knowledge forgetting test, the order of the test questions was changed, and the participants answered them again.

### 3.4 Experimental process

**Figure 1:**
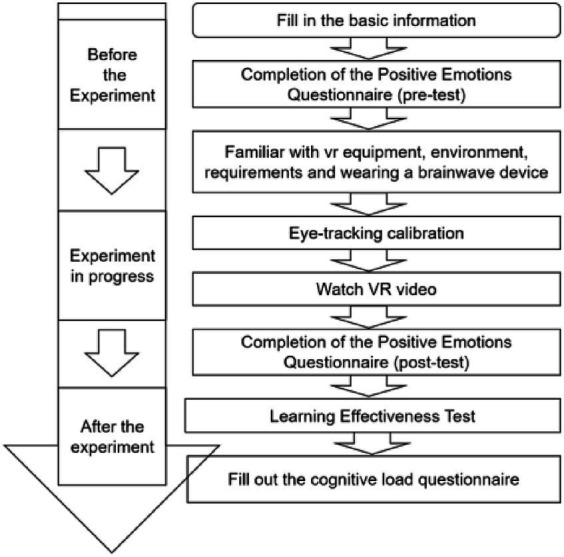
The whole experiment process.

The entire experiment was conducted in an eye-movement laboratory and lasted 25 min. First, the participants provided basic information, completed the pretest questions, and completed the positive emotion scale (pretest) in the reception laboratory. Then, they entered the eye-movement lab and wore the brainwave meter and VR headset. After the participants became accustomed to the equipment, the researcher introduced the experimental procedure. After confirming the participant’s understanding, the participants entered the 360° video learning stage, and their eye-movement trajectories were recorded. After learning, the participants completed the Positive Emotion Scale (posttest), the Cognitive Load Questionnaire, the Retention Test and the Transfer Test. At the end of the experiment, the participants were asked to join a QQ (a social media chat application from Tencent) group to change the order of the learning retention test options; the test was then sent to the participants to reanswer through an online questionnaire a week later as the knowledge forgetting test. The experimental process is shown in Figure 1.

## 4 Results

In this study, there was no significant difference between the pretest-test scores of the four groups of participants (*F =* 0.40, *p* = 0.76). The dependent variables of this study were participants’ positive emotional values (pre-and post-tests); scores on the cognitive load questionnaire; gaze times in the area of interest; average gaze time; learning retention; transfer test; forgetting score; and brainwaves associated with concentration, relaxation, and brain use. The independent variables were cues that acted on different channels. The statistical method is One-way ANOVA. During the experiment, one participant did not have eye movement data recorded, two participants did not complete the online knowledge forgetting questionnaire, and one participant’s brainwave data recording failed. The descriptive statistics of the dependent variables are shown in Table 3.

**Table 3:** Descriptive statistics for the variables.

	Dependent variable	A (*N* = 16, No cues)	B (*N* = 16, Visual cues)	C (*N* = 15, Auditory cues)	D (*N* = 15, Audiovisual cues)
Attention	Average gaze time (ms)	259.19 (39.78)	289.0 (38.14)	255.10 (39.85)	289.80 (46.70)
	Number of gazes in the area of interest	265.50 (34.18)	336.8 (33.45)	281.80 (28.35)	311.90 (45.84)
	Fixed time of first gaze (ms)	210952.62 (3618.96)	207566.30 (346.16)	208689.86 (948.10)	207452.27 (177.40)
Emotion measurement	Positive emotion value (pre-test)	22.67 (5.82)	22.81 (6.69)	23.47 (4.91)	24.07 (3.88)
	Positive emotion value (post-test)	24.33 (4.53)	25.06 (4.60)	24.60 (4.50)	26.40 (3.11)
Cognitive load	Subjective cognitive load	10.69 (1.14)	8.50 (1.27)	10.60 (1.60)	8.53 (1.36)
	Internal cognitive load	13.00 (3.43)	14.13 (4.10)	14.07 (3.47)	14.60 (3.56)
	External cognitive load	8.50 (3.25)	6.19 (2.93)	7.87 (3.11)	5.67 (2.13)
	Associated cognitive load	24.88 (6.61)	26.94 (4.11)	25.07 (4.32)	26.07 (5.34)
Content knowledge tests	Learning retention	6.94 (1.69)	9.50 (1.79)	6.60 (1.45)	9.60 (2.61)
	Learning transfer	1.44 (1.15)	1.50 (1.27)	1.60 (0.91)	2.07 (1.58)
	Knowledge forgetting	4.33 (1.23)	4.38 (0.96)	3.87 (1.06)	4.93 (0.92)
Brainwave data	Concentration	46.81 (3.73)	53.99 (4.67)	43.37 (4.79)	50.36 (5.29)
	Relaxation	51.82 (4.56)	57.57 (7.27)	51.467 (5.48)	52.459 (6.45)
	Brain use	−19.23 (8.04)	−38.70 (20.82)	−17.59 (5.48)	−43.30 (10.59)

Standard deviation and mean values are shown inside and outside the parentheses, respectively-M (SD), and the results are rounded to two decimal places.

### 4.1 Attention allocation

**Figure 2:**
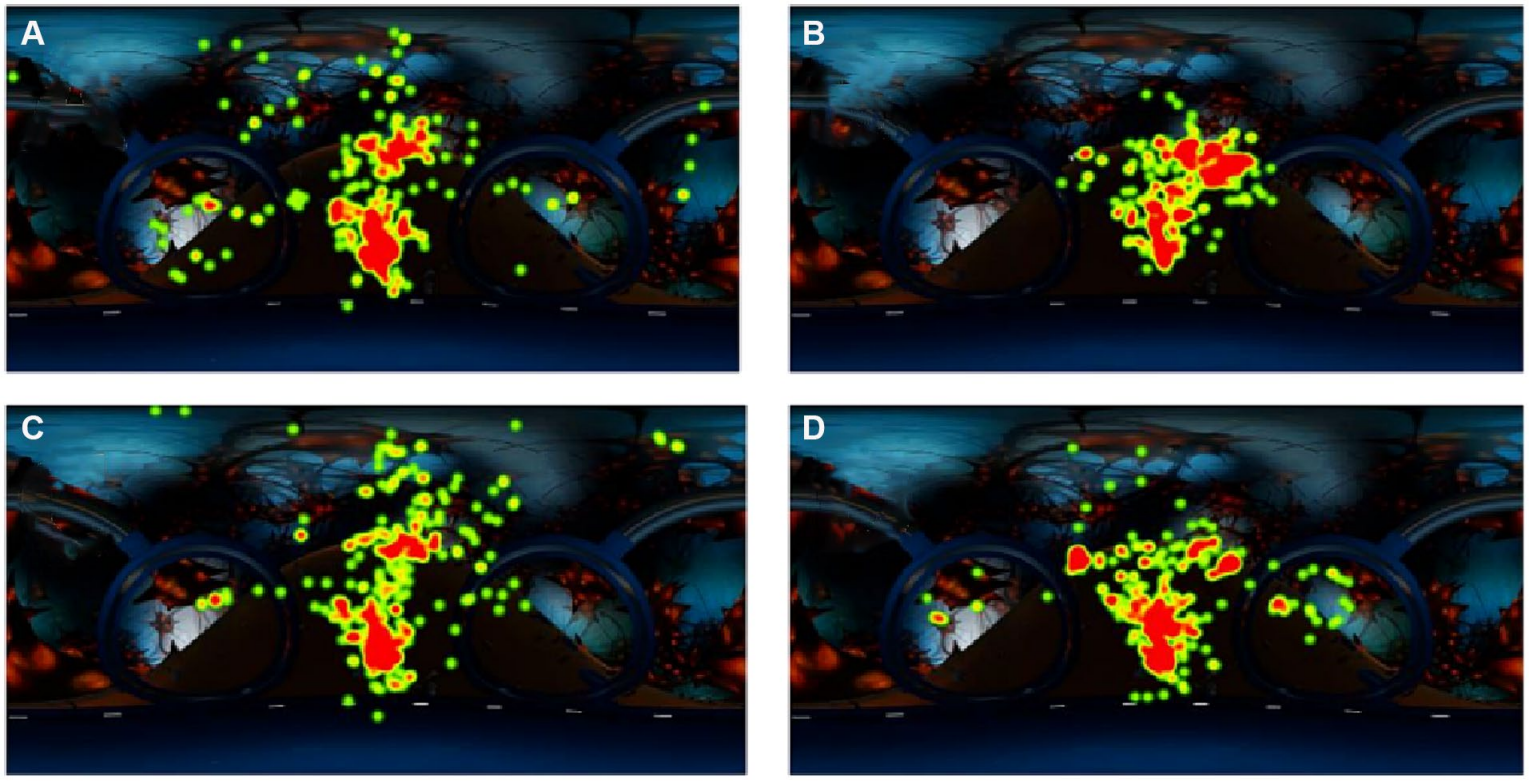
Map of the hot zone of each group’s gaze [Group **(A)**: no cue, Group **(B)**: visual cues, Group **(C)**: auditory cues, Group **(D)**: audiovisual cues].

In this study, the mean gaze point duration and the number of interest area gazes were selected as dependent variables for the eye movement instrument in terms of visual attention. One-way ANOVA revealed that the cues acting on different channels significantly affected visual attention allocation. The visual and audiovisual cues increased the mean gaze duration (*F =* 3.11, *p* = 0.03). The visual and audio-visual cues increased the number of gaze sessions in the area of interest (*F* = 12.17, *p* < 0.001), but the effect of audio-visual cues was weaker than that of visual cues. The effect of cues was significant for the mean time to the first fixation in the area of interest. The visual and audio-visual cues effectively reduced the time learners spent in visual searching, allowing them to focus on specific learning contents. In contrast, the effect of auditory cues was not significant. The one-way ANOVA for each attention-dependent variable is shown in Table 4 and Figure 2.

**Table 4:** Results of one-way ANOVA for attention.

Dependent variable	Sum of squares (between groups)	Mean square	*F*	Significance
Average gaze time (ms)	15824.006	5274.669	3.108	0.033
Number of gazes in the area of interest	47522.160	15840.720	12.173	0.000
First gaze fixed time (ms)	113087747.391	37695915.79	10.749	0.000

### 4.2 Emotion measurements

For the subjective positive mood questionnaire, the data were analyzed by one-way ANOVA and an independent samples *t*-test before and after the test, and the results are shown in Table 3 (Pretest results *F* = 0.26, *p =* 0.86; posttest results *F =* 0.55, *p =* 0.65; paired values of pretest and posttest *t = −*3.57, *Sig* = 0.001). The data indicated no significant difference between the groups before and after the experiment. There was no significant difference in the participants’ emotions, and the groups’ emotion values changed significantly before and after the experiment. Independent sample paired t tests and one-way ANOVA for the dependent variable of mood in each group in terms of relaxation measured by the objective brainwave meter are shown in Tables 5, 6.

**Table 5:** Results of independent sample paired t tests of positive emotions before and after the experience.

Pairing	t	Degree of freedom	Sig.
Pre-experience Emotional Value – Post-experience Emotional Value	−3.568	61	0.001

**Table 6:** Results of one-way ANOVA for mood.

Dependent variable	Sum of squares (between groups)	Mean Square	*F*	Significance
Positive mood values (pre-test)	22.573	7.524	0.257	0.856
Positive emotion value (post-test)	30.540	10.180	0.554	0.647

### 4.3 Cognitive load

The subjective questionnaire revealed that cues acting on different channels had a significant effect on learners’ subjective mental effort (*F =* 12.98, *p* < 0.001). Multiple comparison analyses revealed that the visual and audiovisual cues reduced learners’ subjective mental effort in the 360° video environment, and the effect of audiovisual cues on subjective mental effort was comparable to that of visual cues. In contrast, the auditory cues had no significant effect on subjective mental effort. Moreover, the subjective questionnaire revealed no significant effect of cues acting on different channels on intrinsic cognitive load (*F =* 0.54, *p* = 0. 66) or associative cognitive load (*F* = 0.53, *p* = 0.66) and a significant effect on external cognitive load (*F* = 3.35, *p* = 0.03). Least significant difference (LSD) multiple comparison analysis revealed that the visual and audio-visual cues effectively reduced the external cognitive load. The results of the one-way ANOVA for the dependent variable of cognitive load in each group are shown in Table 7.

**Table 7:** Results of one-way ANOVA for cognitive load.

Dependent variable	Sum of squares (between groups)	Mean Square	*F*	Significance
Subjective mental effort	70.326	23.442	12.977	0.000
Intrinsic cognitive load	21.459	7.153	0.536	0.660
External cognitive load	84.238	28.079	3.354	0.025
Associated cognitive load	43.317	14.439	0.534	0.661

### 4.4 Academic performance

The effect of cues was significant for learning retention performance (*F* = 10.76, *p* < 0.001). The visual and audio-visual cues contributed significantly to knowledge retention, while the effect of auditory cues was not significant. In terms of learning transfer performance, the cue effect was nonsignificant (*F =* 0.80, *p* = 0.50), indicating that cues acting on different channels did not affect knowledge transfer. On the online knowledge forgetting test after one week, there was no significant difference in the scores of the four groups (*F* = 2.48, *p* = 0.07). The results indicated that the visual and audio-visual cues contributed to knowledge transient retention but had no significant effect on knowledge transfer or long-term memory. The one-way ANOVA results for each group’s learning performance are shown in Table 8.

**Table 8:** Results of one-way ANOVA for academic performance.

Dependent variable	Sum of squares (between groups)	Mean Square	*F*	Significance
Learning retention	120.250	40.083	10.756	0.000
Learning Transfer	3.723	1.241	0.796	0.501
Knowledge Forgetting	8.188	2.729	2.475	0.071

### 4.5 Brainwave data

Regarding attention, the cues with different roles significantly affected attention (*F* = 14.94, *p* < 0.001). The data were analyzed by multiple comparisons, which revealed that the visual and audiovisual cues increased implicit attention, the visual cues were the most effective, and the auditory cues decreased implicit attention.

Regarding relaxation, the effect of cues was significant according to one-way ANOVA (*F* = 3.51, *p* = 0.02). The visual cues significantly increased relaxation after multiple comparisons, but auditory and audiovisual cues had no significant effect on relaxation. Perhaps the reason for this result is that in 360° videos, learners receive mismatched information from the auditory and visual channels, while the 360° video narration and scenes are constantly changing linearly, making it more challenging to process the information and causing learners to be nervous or upset. Visual cues can more easily draw learners’ attention to the relevant areas, reduce learners’ tension, and enhance learners’ relaxation. Audiovisual cues may not significantly affect relaxation due to the directional effect of auditory cues, which mitigates the effect of visual cues on relaxation.

Regarding brain use, objective brainwave meter measurements also showed that cues acting on different channels significantly affected the learners’ cognitive load (*F* = 11.86, *p* < 0.001). Multiple comparisons showed that the visual and audiovisual cues effectively reduced the learners’ cognitive load, while the auditory cues had no significant effect. The results of one-way ANOVA for each group of brainwave data are shown in Table 9.

**Table 9:** Results of one-way ANOVA for brainwave data.

Dependent variable	Sum of squares (between groups)	Mean square	*F*	Significance
Attention	970.222	323.407	14.935	0.000
Relaxation	385.30	128.435	3.506	0.021
Brain use	7964.650	2654.883	39.331	0.000

## 5 Conclusion analysis and discussion

The current study investigated the effects of cues acting on different channels in 360° videos on learning declarative knowledge and the underlying mechanisms involved. The results showed that cues acting on different channels affected learners differently. Visual and audiovisual cues effectively guide learners’ attention to the related learning content, reduce the external cognitive load during learning, and improve learning retention performance. However, they have no significant effect on knowledge transfer or long-term memory. Auditory cues increase the number of times learners attend to related learning content, but they do not affect the duration of attention. Auditory cues distract implicit attention during learning and have no significant impact on the acquisition of relevant learning content.

### 5.1 Effects of the embedding cues of different channels in 360° videos on attention

Visual and audiovisual cues effectively direct learners’ visual attention, but auditory cues have no significant effect on visual attention. Visual and audiovisual cues increase implicit attention, with visual cues being the most effective, while auditory cues distract implicit attention.

For the effect of visual cues, in 360° video immersive virtual reality environments, cues (arrows and annotations) can make specific learning content more salient than other content. Visual cues can effectively direct visual attention to learning content (Liu et al., 2022); prevent spatial selection errors, i.e., align visual information with speech narration; and increase learners’ attention. This explanation is supported by the fact that visual cues effectively reduce learners’ visual search time for specific learning content, as shown by the first fixation time in the area of interest in this experiment.

Auditory cues guide learners on where to look in the virtual reality environment. However, auditory cues themselves do not provide content-related information. The presentation time is short, so when auditory cues direct learners’ visual attention to the relevant learning content area, they still need to rely on a visual search to complete the selection of relevant learning information. The lack of support provided by visual cues may result in redundancy between auditory channel cues and narration. Without the support of visual cues, information in the auditory channel and narration may be redundant. Inconsistency between visual information and video narration causes time selection errors and distracts learners’ implicit attention. Thus, in a previous study, auditory cues alone in 360° videos were shown to increase the difficulty of accessing information in a virtual reality environment and to distract learners’ implicit attention, as confirmed by Feintuch et al. (2006).

For the effect of audiovisual cues, in 360° video immersive learning environments, visual information is dominant, and when auditory and visual information cues are available, visual cues are more dominant; therefore, audiovisual cues significantly affect learners’ attention allocation. Based on Baddeley’s working memory model, the multimedia learning process has a modal effect (Baddeley, 1992), which means that cognitive resources are best used when visual information is simultaneously presented in an auditory manner. However, the results of this study showed that cues in 360° videos had an “inverse modal effect,” and the effect of auditory cues on visual cues was not enhanced. The reason is that there is a cross-channel attentional transfer process from auditory to visual attention or from visual to auditory attention in response to audiovisual cues. In the above process, auditory cues have a distracting effect on implicit attention, so auditory cues do not strengthen the effect of visual cues.

At the same time, some studies have shown a channel effect for cues; cues and attentional content appearing in the same channel accelerate cognitive responses (Turatto et al., 2002). In 360° video immersive virtual reality environments, where the most salient information is presented visually, embedding visual cues (arrows and text) that match the audio narration in the area of the scene that requires attention will make the relationship between visual information and audio narration more salient, which supports organization and integration of information; additionally, the consistency principle of the CTML holds (Mayer, 2005a,b). Eye-movement data suggest that auditory audiovisual cues diminish the effect of guiding visual attention; thus, auditory cues do not enhance the effect of visual cues.

### 5.2 Effects of embedding cues of different channels in 360° videos on emotions

This study revealed no significant moderating effect of cues on positive emotions in 360° videos. 360° videos can stimulate positive emotions in learners before and after the experience, and 360° videos provide an immersive presence to enhance emotional arousal (Shen Xialin et al., 2019) and stimulate positive emotions in learners when learning with 360° video content (Stavroulia et al., 2019).

### 5.3 Effects of embedding cues of different channels in 360° videos on cognitive load

Visual and audiovisual cues reduce learners’ cognitive load (subjective mental effort) when learning with 360° videos, precisely the external cognitive load. Moreover, there is no significant effect on the intrinsic cognitive load or associated cognitive load. Visual cues can guide learners’ attention to relevant information, improve the efficiency and effectiveness of finding necessary information (Ozcelik et al., 2010), and effectively reduce learners’ difficulty accessing learning content in 360° videos.

### 5.4 Effect of the embedding cues of different channels in 360° videos on learning performance

Visual and audiovisual cues improve learning retention performance. Based on Meyer’s finding that virtual reality learning environments do not affect learning performance related to declarative knowledge (Meyer et al., 2019), this study embedded cues in 360° videos and confirmed that the cues were effective at enhancing learners’ acquisition of declarative knowledge learning and retention but had no significant effect on knowledge transfer or long-term memory. This finding indicates that cues modulate learners’ attention in 360° video learning environments and that cues positively influence the cognitive process of selection but not the process of organization and integration. The reason for this may be that the novelty of VR technology brings excitement and fun. Learners pay more attention to the learning environment (Rupp et al., 2016), leading them to distract themselves from the novelty of the video content experience and focus more on surface features of the learning content than on deep processing; therefore, cues do not promote a deeper understanding of the learning content. Clark (1994) argued that differences in teaching methods lead to differences in learning rather than differences in the media itself. Therefore, it is possible to think about different instructional approaches to using 360° video resources to enhance learning performance. In a previous study on the effect of pretraining on learning performance prior to VR experiences, Meyer et al. (2019) found that pretraining improved VR performance on declarative knowledge transfer.

## 6 Shortcomings of the study and outlook

Notably, objective conditions, including the sex ratio of the participants (more females than males), the age of the participants, and the differences between the experimental setting and the actual teaching environment, limited this study; moreover, the experimental material in this study used static stereo audio, and the conclusions obtained from auditory cues are applicable only to the audio of static stereo audio. Therefore, the use of these findings to guide actual teaching and resource development needs to be further explored.

This study has produced new findings on the learning retention effects of 360° videos on declarative knowledge acquisition. Future research should be conducted in the following areas.

1. Exploring the effects of using 360° videos as a teaching modality on learning performance.
2. Exploring boundary conditions such as the method, dynamics, and number of cues embedded in 360° videos.
3. Exploring the effects of cues on other knowledge types or subject knowledge during 360° videos learning.
4. Exploring the effects of cues acting on tactile, olfactory, and other channels on learning performance in virtual reality environments.
5. Exploring the effects of immersive spatial audio 360° videos on learning performance.

## Data availability statement

The raw data supporting the conclusions of this article will be made available by the authors, without undue reservation.

## Ethics statement

The studies involving human participants were reviewed and approved by the Local Ethics Committee of China West Normal University. The participants provided their written informed consent to participate in this study.

## Author contributions

GH: Writing – review & editing. CC: Writing – original draft. YT: Writing – review & editing. RL: Writing – review & editing. HZ: Writing – review & editing. LZ: Writing – review & editing.
